# Timing of BNT162b2 vaccine prior to COVID‐19 infection, influence disease severity in patients with hematologic malignancies: Results from a cohort study

**DOI:** 10.1002/cam4.6397

**Published:** 2023-10-25

**Authors:** Odit Gutwein, Katrin Herzog Tzarfati, Arie Apel, Naomi Rahimi‐Levene, Levy Ilana, Tamar Tadmor, Maya Koren‐Michowitz

**Affiliations:** ^1^ Department of Hematology Shamir Medical Center (Assaf Harofeh) Zerifin Israel; ^2^ Sackler Faculty of Medicine Tel Aviv University Tel Aviv‐Yafo Israel; ^3^ Hematology Unit Bnai Zion Medical Center Haifa Israel; ^4^ Faculty of Medicine Technion‐Israel Institute of Technology Haifa Israel

**Keywords:** COVID‐19, COVID‐19 vaccine, hematologic malignancies, Omicron

## Abstract

The COVID‐19 pandemic continues to pose challenges to the treatment of hemato‐oncology patients. Emergence of COVID‐19 variants, availability of vaccine boosters and antiviral treatments could impact their outcome. We retrospectively studied patients with hematologic malignancies and confirmed COVID‐19 during the Omicron outbreak. Of 116 evaluated patients, 16% developed severe or critical COVID‐19. Diagnosis of chronic lymphocytic leukemia (CLL) was significantly associated with severe COVID‐19 (*p* = 0.01). The vaccine effectiveness was related to the timing of the vaccine, with patients who received a mRNA vaccine within 7–90 days prior to COVID‐19 being less likely to develop severe disease compared to all other patients (*p* = 0.019). There was no correlation between disease severity and antiviral therapies. Importantly, 45% of patients undergoing active hematological treatment had to interrupt their treatment due to COVID‐19. In conclusion, patients with hematologic malignancies are at a considerable risk for severe COVID‐19 during the Omicron outbreak, with patients with CLL being the most vulnerable. mRNA vaccines have the potential to protect hematological patients from severe COVID‐19 if administered within the previous 3 months. Hematological treatment interruption is a frequent adverse outcome of COVID‐19 infection.

## Abstract 2

This study investigated the impact of COVID‐19 on patients with hematologic malignancies during the Omicron outbreak. Of the 116 patients evaluated, 16% developed severe or critical COVID‐19. chronic lymphocytic leukemia (CLL) was found to be significantly associated with severe COVID‐19, while mRNA vaccines administered within 7–90 days prior to COVID‐19 reduced the likelihood of severe disease. Additionally, 45% of patients undergoing active hematological treatment had to interrupt their treatment due to COVID‐19.

## 1 INTRODUCTION

Since the emergence of the COVID‐19 pandemic in 2020, patients with hematologic malignancies have been recognized as one of the most vulnerable groups for COVID‐19 severe disease and death.^1^, ^2^ Vaccination against COVID‐19 is strongly encouraged in hemato‐oncology patients. However, the vaccine‐induced immune response is significantly lower compared to controls. This is especially observed in specific subpopulations, including chronic lymphocytic leukemia (CLL) patients and in patients that received CD20 monoclonal antibodies during the last 12 months.^3^, ^4^, ^5^ Booster doses of the vaccine were introduced to improve vaccine efficiency, resulting in enhancement of the immune response in some but not all hematological malignancies patients.^3^, ^6^, ^7^, ^8^ Multiple SARS‐CoV‐2 variants have emerged during the pandemic, changing the original disease course, mortality and infectivity rates. At the end of 2021/beginning of 2022, the Omicron variant (SARS‐CoV‐2, B.1.1.529) become dominant, characterized by high infectivity, vaccine “escape” capability, and lower risk of adverse outcomes compared with previous variants.^9^, ^10^, ^11^ Concurrently, several antiviral drugs for patients at high risk of progression to severe disease, were approved.^12^ A fourth booster dose of the BNT162b2 vaccine for high‐risk populations was approved in Israel in December 2021.^13^

We aimed to discern the outcome of patients with hematologic malignancies during the Omicron outbreak, when the fourth vaccine dose and antiviral drugs were available.

## 2 METHODS

This is a retrospective study including patients aged ≥18 years with hematological malignancies and COVID‐19 infection during the Omicron outbreak in Israel. Data were collected for patients who contracted COVID‐19 starting from January 2022, which coincided with the emergence of the Omicron outbreak in Israel.^14^ Patients were recruited from two medical centers. Medical records were reviewed and only patients with confirmed diagnosis (by real‐time polymerase chain reaction test [PCR] or an institutional supervised rapid antigen test) were enrolled. Hematologic malignancy diagnoses were according to the World Health Organization (WHO) classification, revised 4th edition.^15^ Patient with aggressive malignancies in complete response ≥3 months following chemotherapy were not included in the analysis unless received anti‐CD20 antibodies within the preceding 12 months. COVID‐19 severity was classified according to the National Institute of Health guidelines.^16^ In this report, we use the term “Severe COVID‐19 Disease” to encompass severe and critical cases of COVID‐19.

### 2.1 Statistical analysis

Categorical variables are described as frequencies and percentages. Continuous variables were evaluated for normal distribution using a histogram and are reported as median and interquartile range (IQR). Chi‐square and Fisher's exact tests were used to compare categorical variables between groups, and the Mann–Whitney test was applied to compare continuous and ordinal variables. All statistical tests were two‐sided, and statistical significance was set at *p* < 0.05. Statistical analysis was performed using the SPSS statistical software (IBM SPSS Statistics for Windows, version 28, IBM Corp.,2021).

## 3 RESULTS

### 3.1 Patients' characteristics

We identified 116 patients with hematologic malignancies and confirmed COVID‐19 diagnosis between January 2022 and April 2022. Patients were enrolled from two medical centers: 97 (84%) from Shamir Medical Center (SMC) and 19 (16%) from Bnai Zion Medical Center (BZMC). Diagnosis of COVID‐19 occurred between January 02, 2022 and April 30, 2022, and January 10, 2022 and February 05, 2022 at SMC and BZMC, respectively.

Characteristics of enrolled patients are shown in Table 1. The median age was 72 years (IQR 61–79) and 52% of the patients were males. Diagnoses included lymphoma (non‐ Hodgkin lymphoma and Hodgkin lymphoma) (*n* = 33, 28%), CLL (*n* = 22, 19%), plasma cell neoplasms (*n* = 24, 21%), and myeloid neoplasms (*n* = 37, 32%). One or more comorbidities were reported in 80% of patients, most commonly hypertension (49%), cardiovascular disease (19%), and diabetes mellitus (16%). At the time of COVID‐19, 13% of the patients were treatment‐naïve, 61% were on active hematological treatment, and 30% were previously treated for hematological diseases. Active treatments are listed in Table 1. Fifteen patients (15%) received anti‐CD20 antibodies within 12 months before COVID‐19 diagnosis; among those, five patients were on active anti‐CD20 treatment. Two patients had previously received CAR‐T cell therapy.

**TABLE 1:** Patient characteristics.

Variable	Total *N* = 116
Gender M (%)	60 (52)
Age, years, median (IQR)	72 (61–80)
Comorbidities *N* (% of evaluable)	
Hypertension	57 (49)
Cardiovascular disease	28 (24)
Diabetes mellitus	20 (17)
C hronic lung disease	9 (8)
Chronic kidney disease	7 (6)
Obesity	6 (5)
Smoking	7 (6)
Other comorbidities^a^	11 (10)
Diagnoses *N* (%)	
Lymphoma	33 (28)
Aggressive NHL	13 (11)
Indolent NHL	14 (12)
Classical HL	6 (5)
CLL	22 (19)
Plasma cell neoplasms	24 (21)
Myeloid neoplasms	37 (32)
AML	5 (4)
MDS	5 (4)
PV/ET	9 (8)
MF	7 (6)
CML	11 (10)
Treatment status *N* (%)	
Treatment naïve	15 (13)
Previously treated	30 (26)
On active treatment	71 (61)
Active treatment^b^ *N* (%)	
Chemotherapy	13 (11)
Anti‐CD20	5 (4)
BTKi	5 (4)
BCL2i	4 (3)
IMIDs	23(20)
Proteasome inhibitors	10 (9)
Daratumumab	6 (5)
HMA	5 (4)
Roxulitinib	6 (5)
Hydroxyurea	7 (6)
BCR ABL TKI	7 (6)
Steroids	23 (20)

Abbreviations: AML, acute myeloid leukemia; BCL2i, B‐cell lymphoma 2 inhibitors; BTKi, bruton tyrosine kinase inhibitors; CLL, chronic lymphocytic leukemia; CML, chronic myeloid leukemia; ET, Essential thrombocythemia; HL, Hodgkin lymphoma; HMA, hypomethylating agents; IMIDs, immunomodulatory drugs; MDS, myelodysplastic syndrome; MF, myelofibrosis; NHL, non‐Hodgkin lymphoma; PV, polycythemia vera; TKI, tyrosine kinase inhibitor.

^a^ Other comorbidities included: 3 chronic liver disease, 1 mental retardation, 1 carcinoma of breast, 4 comorbidity not specified

^b^ Alone or in combinations.

### 3.2 COVID‐19 disease and outcome

The degree of COVID‐19 was categorized as asymptomatic in 10% and mild to moderate in 74%. Altogether, 16% (*n* = 18) of patients were categorized as having Severe COVID‐19 Disease (severe illness in 6% and critical illness in 10%). Thirty‐one percent of patients were hospitalized during COVID‐19. Hospitalization was due to COVID‐19 in 21% of patients and for other reasons in 10% of patients.

Out of the 116 patients studied, 78% contracted COVID‐19 between January and February 2022 and 22% between March and April 2022. Severe COVID‐19 Disease cases were reported only during the first period. None of the patients who contracted COVID‐19 during March–April 2022 had Severe COVID‐19 Disease, although two patients were admitted for COVID‐19.

Thirty patients who were receiving active hematological treatment at the time of COVID‐19 had to interrupt their treatment due to COVID‐19 (45% of evaluable patients). The median time off treatment was 10 days (IQR 7–14 days).

Tables 2 and S1 summarize the differences between patients with Severe COVID‐19 Disease and non‐severe disease. Severe COVID‐19 Disease was more commonly associated with older age (>65 years old), more than one comorbidity, and cardiovascular disease. Information on COVID‐19 specific symptoms was available in 89 participants. The most commonly reported symptom was cough (59%), followed by fever (53%), dyspnea (25%), and myalgia (23%). Patients with Severe COVID‐19 Disease were significantly more likely to experience fever, cough, and dyspnea compared to patients with non‐severe disease.

**TABLE 2:** Comparison of patients according to COVID‐19 severity.

Variable	Total	Non‐Severe COVID‐19	Severe COVID‐19	*p*
	*N* = 116 N/evaluable subjects (%)	*N* = 98 N (% of evaluable)	*N* = 18 N (% of evaluable)	
Gender‐Male	60/116 (52)	48 (49)	12 (67)	0.168
Age > 65 years	75/114 (66)	59 (61)	16 (89)	**0.024**
Diagnosis				
CLL	22/116 (19)	**14 (14)**	**8 (44)**	**0.010** ^a^
Lymphoma	33/116 (28)	30 (31)	3 (17)	
Plasma cell neoplasms	24/116 (21)	19(19)	5 (28)	
Myeloid neoplasms	37/116 (32)	**35(36)**	**2 (11)**	
On active treatment	71/116 (61)	61 (62)	10 (56)	0.592
Number of comorbidities				
0–1 comorbidities	75 (66)	69 (72)	6 (35)	**0.003**
2–4 comorbidities	38 (35)	27 (28)	11 (65)	
Vaccinated	106/116 (91)	90 (92)	16 (89)	0.653
Non‐Vaccinated	10/116 (9)	8 (8)	2 (11)	
Booster Dose^b^	88/114 (77)	74 (77)	14 (78)	>0.99
No Booster Dose	26/114 (23)	22 (23)	4 (22)	
Median Days from last vaccine dose	129 (IQR 35–172)	129 (IQR 33–171)	122 (IQR 29–176)	0.692
Last vaccine dose between 7 and 90 days	27/106 (26)	27 (29)	0(0)	**0.019**
Antiviral drugs (In the ambulatory and hospital setting).	51/116 (44)	43 (44)	8 (44)	0.964
Antiviral drugs (In the ambulatory setting/admission for other reasons)^c^	45/116 (39)	40 (40)	5 (28)	0.297
COVID‐19 January February 2022	91/116 (78)	73 (75)	18 (100)	0.012
COVID‐19 March–April 2022	25/116 (22)	25 (24)	0 (0)	

Abbreviation: CLL, chronic lymphocytic leukemia.

Statistically significant (*p* < 0.05) values are indicated in bold.

^a^ Comparison between COVID‐19 severity and subcategories of hematological diagnoses, CLL is significantly associated with Severe COVID‐19 Disease and myeloid neoplasms are negatively correlated with Severe COVID‐19 disease

^b^ After third or fourth booster vaccine dose

^c^ Antiviral drugs administered in the ambulatory setting or in hospital while admitted for other reasons than COVID‐19, not including antiviral drugs given to patients hospitalized with mild‐moderate COVID‐19.

Patients with CLL had a significantly higher risk of developing severe COVID‐19 disease, while patients with myeloid neoplasms had the lowest risk. (*p* = 0.010).

Compared to patients with non‐severe disease, patients with Severe COVID‐19 Disease had lower hemoglobin and platelet values (*p* < 0.0001 and *p* = 0.008, respectively). There was no significant difference in absolute lymphocyte count (ALC), absolute neutrophil count (ANC), total globulin, and lactate dehydrogenase (LDH) values between patients with or without Severe COVID‐19 Disease.

Active hematological treatment was not associated with Severe COVID‐19 Disease. Further analysis according to the number of treatment lines or specific treatment categories did not show an association with Severe COVID‐19 Disease (Table S1), including treatment with anti‐CD20 antibodies within the preceding 12 months.

Most of the patients in this cohort (*n* = 106, 91%) were vaccinated with the BNT162b2 mRNA vaccine, 2, 3, and 4 doses in 12%, 44%, and 33%, respectively. The median time from the last vaccine dose to the current COVID‐19 was 129 days (IQR 35–172). There were no associations between Severe COVID‐19 Disease and vaccinations status (vaccinated compared to unvaccinated) or the number of given doses (up to two compared to three and four vaccination doses). However, patients who received a vaccine dose between 7 and 90 days prior to COVID‐19 infection were significantly less likely to develop Severe COVID‐19 Disease (27/98 compared to 0/18 for patients vaccinated within 7–90 days and other patients, *p* = 0.019). There was no significant association between vaccination between 7 and 180 days prior to a COVID‐19 infection and Severe COVID‐19 Disease (*p* = 0.108). Eight patients received their last vaccine dose within 7 days before the COVID‐19 infection (range 2–6 days); in that group, three patients had Severe COVID‐19 Disease.

Thirty‐eight patients were previously tested for anti SARS‐CoV‐2 IgG antibodies following vaccination and 18 patients (48% of patients tested) had a seronegative response. The serological response to the previous vaccine did not correlate with disease severity.

Antiviral COVID‐19 therapies were given in a proportion of patients (44%). The indication for antiviral COVID‐19 therapies was the prevention of disease progression in non‐severe COVID‐19 high‐risk patients. The antiviral treatment could be administered within 5 days of disease onset. Treatments included nirmatrelvir/ritonavir (*n* = 35, 69% of treated patients), molnupiravir (*n* = 13, 25% of treated patients), and short‐course remdesivir (*n* = 3, 6% of treated patients). Antiviral drugs were administered to 37 patients in an ambulatory setting, to eight patients during hospitalization for reasons other than COVID‐19, and to six patients during hospitalization for mild to moderate COVID‐19. There was no association between antiviral treatments in the entire cohort and Severe COVID‐19 Disease. We reanalysis the effect of antiviral drugs when considering only those administered in an outpatient setting or during hospitalization for other reasons and not for COVID‐19. Results showed a trend towards higher proportion of antiviral drugs treatment in non‐severe cases (40% non‐severe vs. 28% severe), but this difference was not statistically significant (*p* = 0.297). There was no significant difference in the use of antiviral drugs between the two study periods: 39 out of 92 patients (42%) received antiviral treatment in January and February, compared to 12 out of 24 patients (50%) in March and April (*p* = 0.64).

Altogether, seven patients (6%) died due to COVID‐19, with a median time of 16 days (range 4–82 days) from COVID‐19 diagnosis.

## 4 DISCUSSION

The challenge in caring for patients with hematologic malignancies during the COVID‐19 pandemic is to integrate adequate hematological treatment with protection from COVID‐19 during a dynamic pandemic driven by variants. In this study, we evaluated a cohort of patients with hematologic malignancies who contracted COVID‐19 during the Omicron outbreak. Our cohort included the entire spectrum of chronic and aggressive lymphoid and myeloid malignancies, with 61% of patients on active treatments. In this cohort, 21% of patients were hospitalized for COVID‐19, 16% developed severe or critical illness, and 6% died of COVID‐19. These are improved outcomes compared to reported outcomes in pre‐Omicron studies.^2^, ^17^, ^18^ Similar results were shown in additional studies conducted during the Omicron outbreak, which reported hospitalization rates of 26%–43% and mortality rates of 4%–12% in cohorts that included hemato‐oncology patients.^19^, ^20^, ^21^, ^22^, ^23^

We evaluated factors that were associated with COVID‐19 disease severity, using the National Institute of Health criteria. This classification incorporates respiratory parameters to categorize the COVID‐19 status. Hospitalization rates were not chosen as the primary outcome because we recognized that hemato‐oncology patients are frequently hospitalized regardless of COVID‐19,^24^ and a distinction between COVID‐19‐related admission and hematology‐related admission is sometimes cumbersome, especially for patients who contracted COVID‐19 in the hospital.

CLL diagnosis is associated with severe COVID‐19 outcomes. Immunodeficiency related to the disease and treatments, as evident in an inadequate response to COVID‐19 vaccination, explains the susceptibility of CLL patients to COVID‐19‐related complications, as reported across all stages of the COVID‐19 pandemic.^21^, ^25^, ^26^, ^27^, ^28^ Our results further emphasize the vulnerability of patients with CLL and the importance of taking multiple measures to avoid adverse outcomes of COVID‐19.

Our analysis suggests that patients with myeloid neoplasms are less susceptible to severe COVID‐19 than patients with other hematologic malignancies. This could be attributed to the inclusion of patients with chronic myeloid leukemia, polycythemia vera, and essential thrombocytosis in this category, a subpopulation of patients with a lower rate of COVID‐19‐related complications.^29^, ^30^

Active hematological treatment or specific treatment groups were not correlated with COVID‐19 severity in this report. A specific group of interest is patients who had received an anti‐CD20 antibody in the last 12 months because of the significantly reduced humoral response to the vaccine documented in this population.^8^ Here, we did not find a correlation between Severe COVID‐19 Disease and exposure to anti‐CD20 antibodies. Lack of correlation between anti‐CD20 therapy and COVID‐19 outcome was also noticed in other studies conducted during the Omicron outbreak, which included patients with lymphoproliferative malignancies.^19^, ^20^, ^21^ One study of hematologic patients during the Omicron wave described high mortality rate in patients treated with anti‐CD20 antibodies, but the statistical significance of this finding was not reported One potential explanation for the lack of correlation between anti‐CD20 therapy and COVID‐19 outcome during the Omicron outbreak could be a sustained humoral response from vaccination given before initiating the therapy, combined with preserved cellular response.^5^, ^8^

Our cohort was highly vaccinated, with more than 90% of patients being vaccinated with the BNT162b2 mRNA vaccine, and 77% of patients receiving a third or fourth dose. Considering evidence regarding the ability of the Omicron variant to evade immunity from previous vaccines,^9^, ^31^ we were interested in whether vaccine status affected COVID‐19 severity. We found that being previously vaccinated or the number of given vaccines did not correlate with COVID‐19 severity. Since the immunological protection of the BNT162b2 mRNA vaccine begins 7 days after vaccination and decreases after 90 days,^32^, ^33^, ^34^ we compared COVID‐19 severity between 2 periods: first 7 days and 90 days from vaccination, and 7–90 from vaccination. Indeed, we found lack of Severe COVID‐19 Disease in patients receiving the vaccine within the period of highest effectiveness (7–90 days). Our findings on the time‐related effectiveness of the COVID‐19 vaccine in patients with hematologic malignancies are supported by data from other populations. Several studies conducted during the Omicron surge in the general population, demonstrated the benefit of a booster dose (third or fourth) when administered in close proximity to the infection but effectiveness loss with time.^35^, ^36^, ^37^, ^38^ In an analysis of immunocompromised patients during the Omicron predominance, vaccine effectiveness against COVID‐19‐related hospitalization was highest between 7 and 89 days after the booster dose,^39^ which is in accordance with our results. Studies in patients with lymphoproliferative disease did not find a correlation between vaccine status and COVID‐19‐related outcomes, although the timing of vaccination was not specified in these studies.^20^, ^21^

For the first time during the pandemic, concurrent with the Omicron surge, oral antiviral drugs were available and indicated to prevent COVID‐19 progression in high‐risk patients. Large “real‐world” studies have shown that antiviral drugs were effective in the general population during the Omicron surge when given at home.^40^, ^41^ In our patient population which represents a “real life” scenario, antiviral drugs were administered in both the ambulatory and hospital settings. We could not show a significant relationship between the use of antiviral drugs and severe COVID‐19. Antiviral drugs, however, were originally studied only in non‐hospitalized COVID‐19 patients and were intended to prevent hospitalizations due to COVID‐19.^42^, ^43^, ^44^ Since hematologic malignancies patients are often hospitalized for indications other than COVID‐19 we reanalyzed the data combining patients hospitalized for non‐COVID‐19 reasons receiving antiviral drugs with the group of ambulatory patients. In this analysis, fewer patients with Severe COVID‐19 had received antiviral drugs compared to those with non‐severe disease. This difference, however, was not statistically significant. Of note, administration of antiviral drugs was also associated with a reduction in adverse outcomes in small groups of solid organ transplant recipients and patients with CLL.^21^, ^45^

Interrupting oncology treatment can have a negative effect on patient outcomes and is therefore undesirable.^46^, ^47^, ^48^ The decision on deferral of hematological treatments in COVID‐19‐positive patients is usually made based on the underlying malignancy and the specific treatment. In this cohort, 45% of patients on active therapy had treatment interruptions at the time of COVID‐19. Although the reason for treatment interruptions were not elaborated in our cohort, those might include COVID‐19 illness and symptoms, need to avoid immunosuppression, drug interactions with antiviral treatments, and infection control considerations. Similar rates (64%) of treatment delays was also reported by Kareff et al., who evaluated oncology and hematology patients receiving cancer‐directed therapies during the Omicron wave.^22^ The long‐term consequences of treatment delays during the pandemic are still unknown; however, interference in treatment plans should be regarded as an adverse outcome of COVID‐19 infection, irrespective of COVID‐19 severity.

Most patients in our study contracted COVID‐19 during the first period of the study (January–February 2022), simultaneously with the peak of COVID‐19 cases in Israel.^14^ The second study period (March–April 2022) was characterized by few cases and no Severe COVID‐19 cases. This could be explained by the dominance of the Omicron BA.2 sub‐variant in the second period, which was reported to be less pathogenic compared to the previous Omicron sub‐variant BA.1.^49^, ^50^

This report had several limitations. The relatively small sample size could have affected our ability to detect more significant findings and perform multivariate regression analysis to reduce the influence of confounding factors. Since the end of February 2022, Evusheld (tixagevimab and cilgavimab) was available for pre‐exposure prophylaxis in severely immunocompromised patients, but we do not have specific data on its use in our group. Indeed, during the period when Evusheld was available, none of the patients in our cohort developed Severe COVID‐19. We did not have sufficient data on SARS‐CoV‐2 IgG antibody levels, which limited our ability to describe the humoral immune response to vaccines and its correlation with COVID‐19 outcomes.

## 5 CONCLUSION

The Omicron outbreak in patients with hematologic malignancies has resulted in a considerable number of Severe COVID‐19 cases. Patients with CLL were especially susceptible to Severe COVID‐19 and represent an extremely high‐risk population. Hematological treatment interruption is a frequent event and should be regarded as an additional adverse outcome of COVID‐19. The mRNA vaccine is effective in patients with hematologic malignancies mainly if given within 90 days prior to Omicron variant infection. Considering a 3‐month period of increased vaccine efficacy may aid in determining the optimal timing for re‐vaccination in extremely high risk populations.

## AUTHOR CONTRIBUTIONS

**Odit Gutwein:** Conceptualization (equal); data curation (equal); formal analysis (equal); investigation (lead); methodology (equal). **Katrin Herzog Tzarfati:** Data curation (equal); methodology (equal). **Arie Apel:** Data curation (equal); investigation (equal). **Naomi Rahimi‐Levene:** Data curation (equal); investigation (equal). **Ilana Levy:** Data curation (equal). **Tamar Tadmor:** Investigation (equal); methodology (equal). **Maya Koren‐Michowitz:** Conceptualization (equal); formal analysis (equal); investigation (equal); methodology (equal).

## ETHICAL APPROVAL

The study was approved by the local Institutional Review Boards, and the need for obtaining informed consent was waived because of the retrospective nature of the study.

## Supporting information


Table S1.

## Data Availability

The information on patients has been abstracted and coded to exclude the personal identification of subjects. Further information is available from the corresponding author upon request.
