# Supplementary material for: Timing of BNT162b2 vaccine prior to COVID‐19 infection, influence disease severity in patients with hematologic malignancies: Results from a cohort study
Source: Cancer Med. 2023 Oct 25;12(21):20503–10. doi: 10.1002/cam4.6397 (PMC10660398; doi:10.1002/cam4.6397)
Supplement: Supplementary file 1 — Table S1. [file CAM4-12-20503-s001.docx]

**Table S1(Supplement)**

**Comparison of Patients according to COVID-19 severity**

| Variable | | Non-Severe COVID-19 | Severe COVID-19 | p |
| --- | --- | --- | --- | --- |
|  |  | N=98  *n (% of evaluable)* | N=18  *n (% of evaluable)* |  |
| Comorbidities | Hypertension | 48 (49) | 9 (50) | 0.937 |
|  | Cardiovascular disease | 19 (19) | 9 (50) | **0.013** |
|  | Diabetes mellitus | 16 (16 | 4 (22) | 0.511 |
|  | Chronic lung disease | 7 (7) | 2 (11) | 0.628 |
|  | Chronic kidney disease | 4 (4) | 3 (16) | 0.074 |
| Number of comorbidities | 0 | 20 (21) | 0 (0) | **0.002** |
|  | 1 | 49 (51) | 6 (35) |  |
|  | 2 | 17 (18) | 7 (41) |  |
|  | 3 | 8 (8) | 3 (18) |  |
|  | 4 | 2 (2) | 1 (6) |  |
| Treatment  Categories ^a^ | Chemotherapy | 12 (12) | 1 (6 | 0.688 |
|  | BTKi and BCL2i | 8 (8) | 1 (6) | 1.000 |
|  | IMIDs, PI, Daratumumab | 21 (21) | 6 (33) | 0.361 |
|  | Treatment for myeloid neoplasms ^b^ | 25 (25) | 2 (11) | 0.236 |
|  | Steroids ^c^ | 19 (19) | 4 (22) | 0.753 |
|  | Anti-CD20 within 12 months | 14 (14) | 1 (6) | 0.461 |
| Number of treatment lines | 0 | 10 (10) | 5 (28) | 0.336 |
|  | 1 | 61 (62) | 8 (44) |  |
|  | ≥2 | 27 (28) | 5 (28) |  |
|  | Previous negative serologic reaction for vaccine | 15 (46) | 3 (6( | 0.653 |
| COVID-19 symptoms | Fever | 34(47) | 13 (77) | **0.030** |
|  | Cough | 38 (53) | 14 (88) | **0.011** |
|  | Dyspnea | 7 (10) | 15 (88) | **0.000** |
|  | Diarrhea | 5 (7) | 1 (7) | 1.000 |
|  | Sore throat | 6 (9) | 0 (0) | 0.583 |
|  | Myalgia | 15 (21) | 4 (29) | 0.727 |
| Baseline laboratory values ^c^ | Absolute Neutrophil Count 10^9/L, median (IQR) | 3.7 (2.3-5.2) | 2.98 (1.1-4.2) | 0.141 |
|  | Absolute Lymphocyte Count 10^9/L, median (IQR) | 1.4 (0.9-2.05) | 1.4 (0.475-22.5) | 0.786 |
|  | Platelet 10^9/L, median (IQR) | 189 (137-260) | 108 (44-207) | **0.008** |
|  | Hemoglobin g/dL, median (IQR) | 12.2 (10.8-13.6) | 9.9 (8.925-11.4) | **0.000** |
|  | Globulin g/L, median (IQR) | 27 (22-30) | 25.35 (19 -31) | 0.485 |
|  | LDH U/L, median (IQR) | 373 (308-457) | 372 (288-476) | 0.870 |
|  | LDH>UNL | 16 (18) | 4 (24) | 0.591 |

^a^ Some patients received combinations of the following treatments. ^b^ Tyrosine kinase inhibitor, Ruxolitinib, Hydroxyurea, Hypomethylating agents. ^c^ Combined with other treatments. ^c^ Baseline laboratory values taken at COVID-19 diagnosis or up to 3 months before COVID-19 diagnosis. Abbreviations: BTKi, bruton tyrosine kinase inhibitors; BCL2i, B-cell lymphoma 2 inhibitors; IMIDs, immunomodulatory drugs; PI, Proteasome inhibitors; LDH, lactate dehydrogenase; UNL, upper normal limit
